# Detecting geospatial patterns of *Plasmodium falciparum* parasite migration in Cambodia using optimized estimated effective migration surfaces

**DOI:** 10.1186/s12942-020-00207-3

**Published:** 2020-04-10

**Authors:** Yao Li, Amol C. Shetty, Chanthap Lon, Michele Spring, David L. Saunders, Mark M. Fukuda, Tran Tinh Hien, Sasithon Pukrittayakamee, Rick M. Fairhurst, Arjen M. Dondorp, Christopher V. Plowe, Timothy D. O’Connor, Shannon Takala-Harrison, Kathleen Stewart

**Affiliations:** 1 Center for Geospatial Information Science, Department of Geographical Sciences, University of Maryland, College Park, 20742 MD USA; 2 Duke Global Health Institute, Duke University, Durham, 27710 NC USA; 3 Institute for Genome Sciences, University of Maryland School of Medicine, Baltimore, 21201 MD USA; 4 Center for Vaccine Development and Global Health, University of Maryland School of Medicine, Baltimore, 21201 MD USA; 5 Armed Forces Research Institute of Medical Sciences, Bangkok, Thailand; 6 Oxford University Clinical Research Unit, Ho Chi Minh City, Vietnam; 7 Department of Clinical Tropical Medicine, Mahidol University, Bangkok, Thailand; 8 National Institutes of Health, Bethesda, MD USA; 9 Mahidol-Oxford Tropical Medicine Research Unit, Bangkok, Thailand

**Keywords:** *Plasmodium falciparum*, Estimated effective migration surfaces, Parasite migration, Malaria elimination

## Abstract

**Background:**

Understanding the genetic structure of natural populations provides insight into the demographic and adaptive processes that have affected those populations. Such information, particularly when integrated with geospatial data, can have translational applications for a variety of fields, including public health. Estimated effective migration surfaces (EEMS) is an approach that allows visualization of the spatial patterns in genomic data to understand population structure and migration. In this study, we developed a workflow to optimize the resolution of spatial grids used to generate EEMS migration maps and applied this optimized workflow to estimate migration of *Plasmodium falciparum* in Cambodia and bordering regions of Thailand and Vietnam.

**Methods:**

The optimal density of EEMS grids was determined based on a new workflow created using density clustering to define genomic clusters and the spatial distance between genomic clusters. Topological skeletons were used to capture the spatial distribution for each genomic cluster and to determine the EEMS grid density; i.e., both genomic and spatial clustering were used to guide the optimization of EEMS grids. Model accuracy for migration estimates using the optimized workflow was tested and compared to grid resolutions selected without the optimized workflow. As a test case, the optimized workflow was applied to genomic data generated from *P. falciparum* sampled in Cambodia and bordering regions, and migration maps were compared to estimates of malaria endemicity, as well as geographic properties of the study area, as a means of validating observed migration patterns.

**Results:**

Optimized grids displayed both high model accuracy and reduced computing time compared to grid densities selected in an unguided manner. In addition, EEMS migration maps generated for *P. falciparum* using the optimized grid corresponded to estimates of malaria endemicity and geographic properties of the study region that might be expected to impact malaria parasite migration, supporting the validity of the observed migration patterns.

**Conclusions:**

Optimized grids reduce spatial uncertainty in the EEMS contours that can result from user-defined parameters, such as the resolution of the spatial grid used in the model. This workflow will be useful to a broad range of EEMS users as it can be applied to analyses involving other organisms of interest and geographic areas.

## Background

Understanding the genetic structure of natural populations provides insight into the demographic and adaptive processes that have affected those populations, such as migration or natural selection. Such information can have important applications in fields such as conservation biology or public health, particularly when integrated with geographic data. For example, geospatial modeling methods have been used to analyze pathogen genetic or genomic data to understand spatial transmission patterns of influenza virus [1–4] and typhoid fever [5], the sources of imported malaria [6] and dengue infections [7], and malaria parasite landscape genetics [8].

Often estimates of population structure are made without regard to the geographic coordinates of sampling locations and then later interpreted in the context of the geographic information. However, approaches have been developed that model both the spatial and genomic data. One such approach, called estimated effective migration surfaces (EEMS) [9, 10], uses genomic data for a species to visualize the spatial contours of migration and diversity for this species for a given study area. The model broadly assumes isolation-by-distance, whereby genetic similarity and geographic distance are negatively correlated, and identifies areas where genetic similarity decays faster than expected for a given geographic distance (low effective migration) and areas where genetic similarity decays more slowly than expected for a given geographic distance (high effective migration). The model output is a map of areas of high and low effective migration or diversity for the study region. An assumption underlying EEMS is that the population structure is consistent with isolation-by-distance and as such, EEMS results represent effective (i.e., relative) rather than absolute migration rates and are likely to capture patterns associated with more historic timescales. It should be noted that the EEMS toolkit has also been expanded to support identity-by-descent approaches and estimations of migration and population-size surfaces for more recent time scales with the MAPS toolkit [11]. For a historic understanding of migration patterns in a region, EEMS is a useful tool and has been used, for example, to understand the population structure of human populations in southern [12] and eastern [13] Africa and in Europe [14], and to visualize barriers and corridors of gene flow associated with human migration in Scandinavia [15] and Peru [16]. EEMS has also been applied to simulate historical gene flow patterns for the gray wolf (*Canis lupus*) [17] and the blunt-nosed leopard lizard *Gambelia sila* [18], and to investigate the genetic diversity of Atlantic Bluefin tuna in the Mediterranean Sea [19]. In this paper, we use EEMS to estimate migration surfaces for *Plasmodium falciparum,* the deadliest human malaria species.

In our previous research, we have applied EEMS as well as approaches based on identity-by-descent to investigate migration patterns and population structure of *Plasmodium falciparum* in the Greater Mekong Subregion [20], an area of emerging multidrug resistance being targeted for malaria elimination [21]. EEMS maps are visually–intuitive and may be useful to malaria elimination programs by identifying defined geographic areas that can be targeted with interventions. However, to be useful for this purpose, it will be important to reduce spatial uncertainty in the EEMS contours that can arise from user-defined parameters, such as the resolution of the spatial grid used in the model. The spatial grid is a grid of regular triangles that covers the study area. Each vertex in the grid represents a *deme*, and EEMS uses the number of demes selected by a user to generate the spatial resolution of its spatial grids. The random selection of the number of demes can result in a high standard deviation among posterior distributions estimated using EEMS and in turn, higher levels of spatial uncertainty in the migration contours generated by EEMS. For example, if the grid is too sparse (i.e., relatively fewer demes), then many sampling locations may be assigned to a single deme, reducing model accuracy through excessive smoothing of genomic differences. On the other hand, if the grid is too dense (i.e. relatively high numbers of demes) spatial uncertainty may result from estimation of parameters for many demes lacking genomic data [20]. In addition, the number of demes included in the analysis has a substantial impact on computing time, with computing time scaling cubically with the number of demes. Researchers typically employ either an average of the results obtained from running multiple MCMC iterations using different numbers of demes to infer migration patterns [9, 17, 22, 23], or apply maximum likelihood values to guide selection of the number of demes [20]. In both cases, users must run EEMS at multiple grid resolutions, which can be time-consuming.

Here we present an approach that utilizes a density clustering algorithm to define genomic clusters, which are then used to determine the optimal maximum length of triangle edges and grid resolution. This workflow provides a systematic method to select the optimal number of demes that will maximize model accuracy and minimize computing time. We tested the optimized workflow by applying it to estimate geospatial patterns of *Plasmodium falciparum* migration in Cambodia and bordering regions of Thailand and Vietnam, and found that migration contours corresponded to estimates of malaria endemicity and geographic properties of the region that might be expected to impact malaria parasite migration.

## Methods

### Study area and data collection

**Fig. 1:**
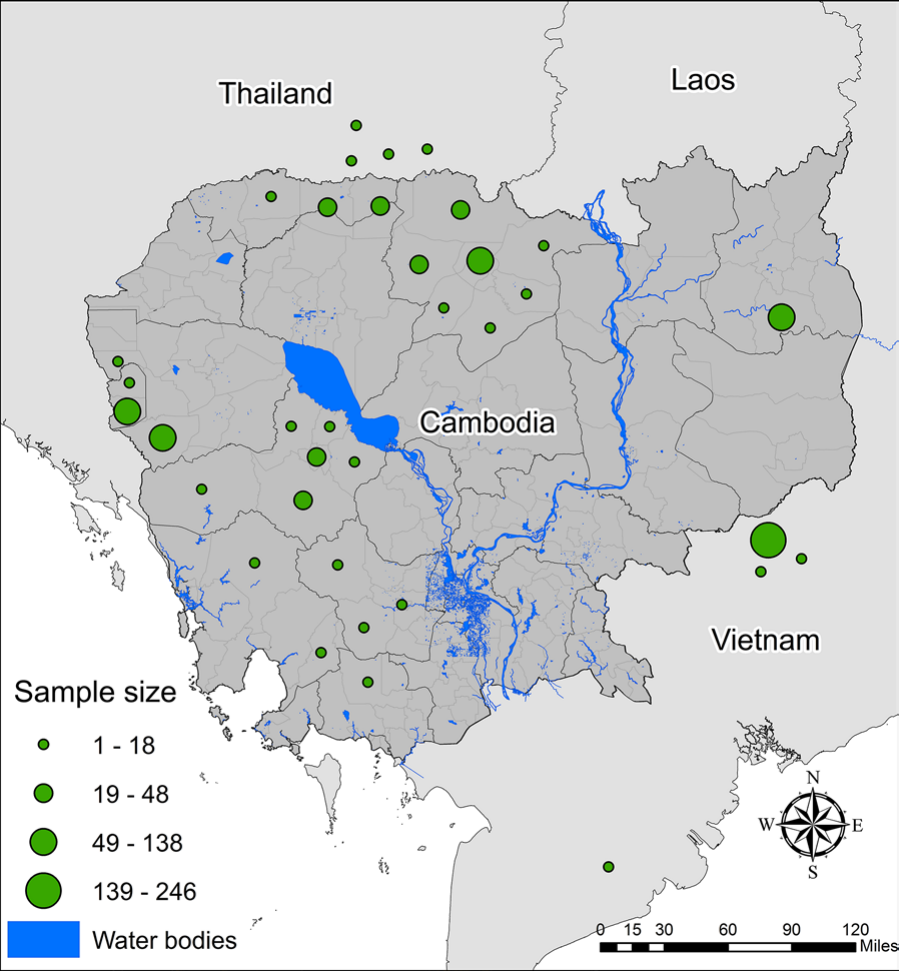
Sampling locations in Cambodia, Thailand and Vietnam

Our approach was tested on a subset of the *P. falciparum* genomic data from our previous publication [20], including 28,496 biallelic, genome-wide SNPs from 1007 samples collected in 35 districts in Cambodia and 8 bordering districts of Thailand and Vietnam between 2008 and 2013 (Fig. 1) [24–28]. SNPs were either called from whole genome sequences generated as part of the MalariaGEN *Plasmodium falciparum* Community Project [29], or, for samples that did not meet quality control criteria for whole genome sequencing or were not part of the Community Project, were genotyped using a *P. falciparum*-specific Nimblegen DNA microarray [30] (NIH Gene Expression Omnibus, Accession number: GSE100704. European Variant Archive, Accession PRJEB28530). The same nucleotide positions typed on the microarray were extracted from whole-genome data for analysis, with missingness cut-offs applied as previously described [20].

### Computing estimated effective migration surfaces

EEMS utilizes a grid of regular triangles that cover the study area. The grid is created using two user-defined parameters, a bounding box that defines the geographic area where gene flow will be modeled, and the number of *demes*, where a deme represents a vertex in the grid. The EEMS toolkit allows the number of demes to vary up to a maximum value of 1000, allowing for different grid resolutions. Genomic data from a given sampling location is assigned to the nearest deme, and the model uses the deme locations to estimate and map effective migration surfaces. EEMS assumes individuals migrate locally between subpopulations (at demes) and that migration rates vary by location. The model also assumes that each subpopulation exchanges migrants only with its neighbors (i.e., a stepping-stone model). For every triangle in the grid, EEMS assigns diversity estimates to demes and migration estimates to triangle edges. Markov chain Monte Carlo (MCMC) methods are employed to estimate both migration and diversity parameters by sampling from their posterior distributions given observed genetic dissimilarities. The matrix of average pairwise genetic dissimilarities is computed using *bed2diffs*, and EEMS is then run using *runeems_snps*: a C ++ implementation of EEMS for SNP data.

For each iteration, two sets of Voronoi tessellations are generated, representing spatial patterns of migration and diversity respectively. These tessellations are generated based on a user-defined value *nseeds* that represents the number of Voronoi cells and that is also assigned to the grids. The two Voronoi tessellations are independent of each other and are updated with a birth/death process since the number of Voronoi cells is initially unknown. A maximum likelihood method is commonly used to adjust estimates of diversity and migration so that simulated genetic dissimilarity rates fit observed genetic dissimilarity in both cases.

### Clustering based on the distribution of *P. falciparum* genomic data

**Fig. 2:**
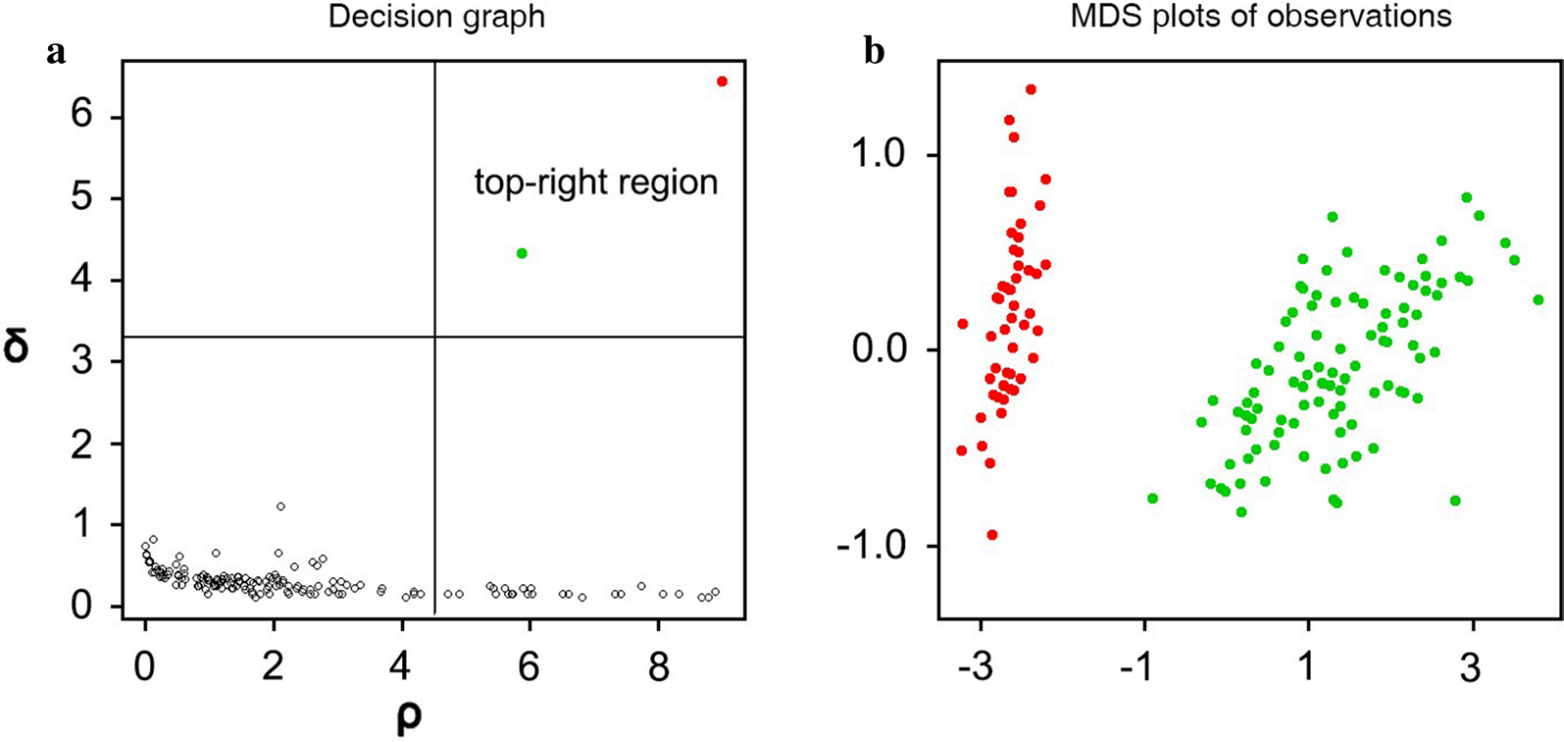
**a** Decision graphs generated from clustering by fast search and find of density peaks [31] and **b** multidimensional scaling graph generated from extracting density peaks

To optimize the grid, we first applied a density clustering algorithm to define clusters based on parasite genomic data [31]. Clustering was performed using *densityClust*, an algorithm that is an improvement on K-means clustering, and is available as an open source package in R [32]. This clustering method did not require prior knowledge of the desired number of clusters and assumed that cluster centers were distinct from points with higher local density and were surrounded by points with low local density. Clustering was performed using the matrix of pairwise genetic dissimilarities generated through the EEMS toolkit, as a measure of genetic distance. A decision graph (Fig. 2a) and a multidimensional scaling graph (Fig. 2b) were generated for all the samples, where the x-axis represented the local density p_*i*_ of sample *i* and the y-axis represented the genetic distance from the nearest points with a higher density δ_i_. The local density p_*i*_ was defined as:

1 $$ {\text{p}}_{i} = \mathop \sum \limits_{j} \chi \left( {d_{ij} - d_{c} } \right) $$

where $\chi \left( x \right) = 1$ if *x *< 0 and $\chi \left( x \right) = 0$ otherwise. *d*_c_ was the cutoff distance and p_*i*_ was equal to the number of samples that were closer than *d*_c_ to sample *i*. δ_i_ was calculated by measuring the minimum distance between the sample *i* and other samples with higher density:

2 $$ {{\delta }}_{i} = \mathop {\text{min} }\limits_{{j:p_{j} > p_{i} }} \left( {d_{ij} } \right) $$

For the sample with the highest density, we assumed δ_i_ = max_j_(*d*_ij_), where δ_i_ would be much greater than the typical nearest neighbor distance only for samples that with the local or global maxima density and cluster centers were recognized as samples for which the δ_i_ value was anomalously large.

The output of the decision graph was used to confirm the number of genomic clusters. Points located in the upper-right quadrant distant from the other points are more likely to be cluster centers (Fig. 2a). All points were treated as cluster centers as long as their *p*_*i*_ and δ_i_ were higher than the mean *p*_*i*_ and δ_i_ value. After the number of cluster centers and hence, the number of clusters was determined, all the samples were assigned to a cluster based on genetic similarity (Fig. 2b).

### Computing the length of triangle edges and determining the number of demes

The genomic clustering results were used to determine the optimal maximum length of triangle edges. For each cluster, a kernel density map was generated based on sample sizes and locations. A natural breaks classification was used to group each kernel density map into binary categories of high and low sample density, respectively. This classification method minimized the average deviation from the class mean and maximized the deviation from the means of the other groups. It also reduced the variance within classes while maximizing the variance between classes. The category representing the highest sample density was selected to determine the spatial distribution of boundaries that represent each cluster and from which triangle lengths could be established. Since the cluster polygons were often irregularly shaped, the topological skeletons of polygons were used to capture polygon shapes. To represent the spatial distance between clusters, the nearest distance between each pair of topological skeletons was computed. Finally, the shortest distance between each pair of clusters was determined and selected to represent the *maximum length* of a triangle edge in the grid. To determine the number of demes that optimizes the grid, this optimized triangle edge length was used as input in an inverse function to the EEMS grid generation function with the result that the edge length of the generated EEMS grid is shorter than the optimized triangle edge length. In this way, both genomic and spatial clustering were used to guide the optimization of triangle sizes and the density of demes, i.e., grid resolution, used for generating *P. falciparum* parasite migration maps.

### Evaluation of model accuracy

While the EEMS toolkit can be used to generate both migration and diversity maps for a bounded region [9], in this study, we focused particularly on migration maps, using genomic data from *P. falciparum*. The EEMS toolkit allows the generation of scatterplots to visualize the correlation between observed and fitted genetic dissimilarity between demes to determine model accuracy for migration maps. Such scatterplots have been used by researchers to evaluate the model accuracy of EEMS contours [33–36]. For each grid density, R^2^ was estimated and compared to determine how migration model accuracy varied by the number of demes.

## Results

**Fig. 3:**
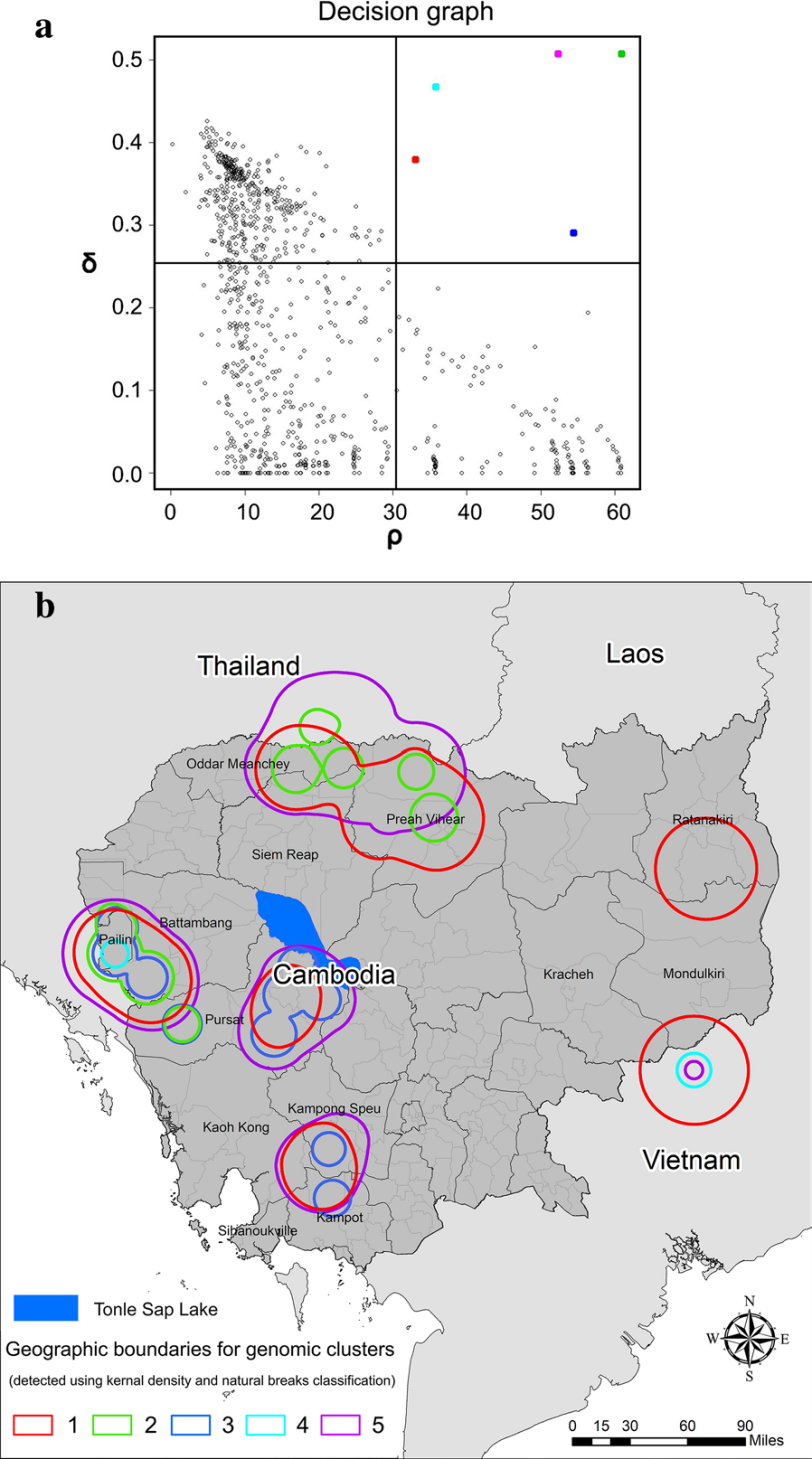
Density-based clustering of genomic data where **a** five genomic clusters that were detected using the decision graph corresponded geographically to **b** six geographic locations (five locations in Cambodia and one in Vietnam)

Applying density clustering to the *P. falciparum* genomic data from Cambodia and bordering sites in Thailand and Vietnam, the decision graph identified five genomic clusters (Fig. 3a). Kernel density analysis was applied to generate a map of these clusters and showed the five genomic clusters occupied six different locations (Fig. 3b). The six locations included, (1) northwestern Cambodia bordering Thailand (Oddar Meanchey and Preah Vihear Provinces), (2) western Pailin Province on the eastern border with Thailand, (3) south of Tonle Sap Lake in Pursat Province, (4) the adjacent region in southeastern Koh Kong Province, southwestern Kampong Speu Province and the northern part of Kampot Province, (5) eastern Cambodia in an area that overlapped southern Ratanakiri Province and northern Mondulkiri Province, and (6) Bu Dop district, Vietnam (Fig. 3b). In Fig. 3a, one genomic cluster (colored red) was identified in all six locations, while another cluster (cyan) was found in only two locations, namely the Pailin District in western Cambodia and northern Bu Gia Map National Park in Vietnam. All five genomic clusters were found in Pailin District, whereas only one genomic cluster (red) was found in eastern Cambodia in the area where Ratanakiri Province borders Mondulkiri Province (Fig. 3b).

### Generating an optimized grid for the study area

The shortest distance between cluster centers computed using the topological skeletons of the clusters was 32.6 km, representing the maximum edge length for each triangle in the EEMS grid. The corresponding number of demes was calculated by setting the longest edge length to this value and using the inverse function as described above. Using our workflow, the optimized grid contained 350 demes (Additional file 1: Figure S1). The MCMC iteration using this grid resolution was 30 million; burn-in was 29 million; and thinning iteration was 9999. The running time was approximately 13 h using 64 CPUs on a Linux high-performance network.

### Evaluating the optimized grid

**Fig. 4:**
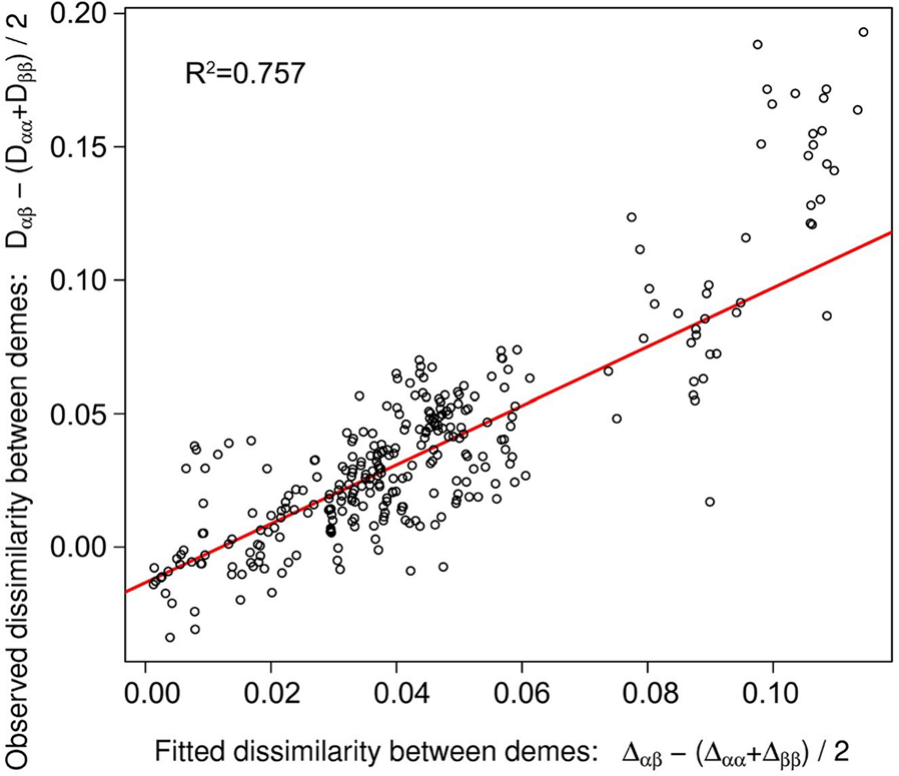
EEMS model accuracy for between-demes using the optimized grid

We investigated migration model accuracy for the optimized grid by examining scatterplots of the observed genetic dissimilarity between demes versus the fitted genetic dissimilarity between demes. The fitted genetic dissimilarly was calculated based on the computed migration between pairs of deme locations. The scatter plots showed a strong linear relationship (R^2^ value was 0.757) between observed and fitted dissimilarity (Fig. 4).

**Fig. 5:**
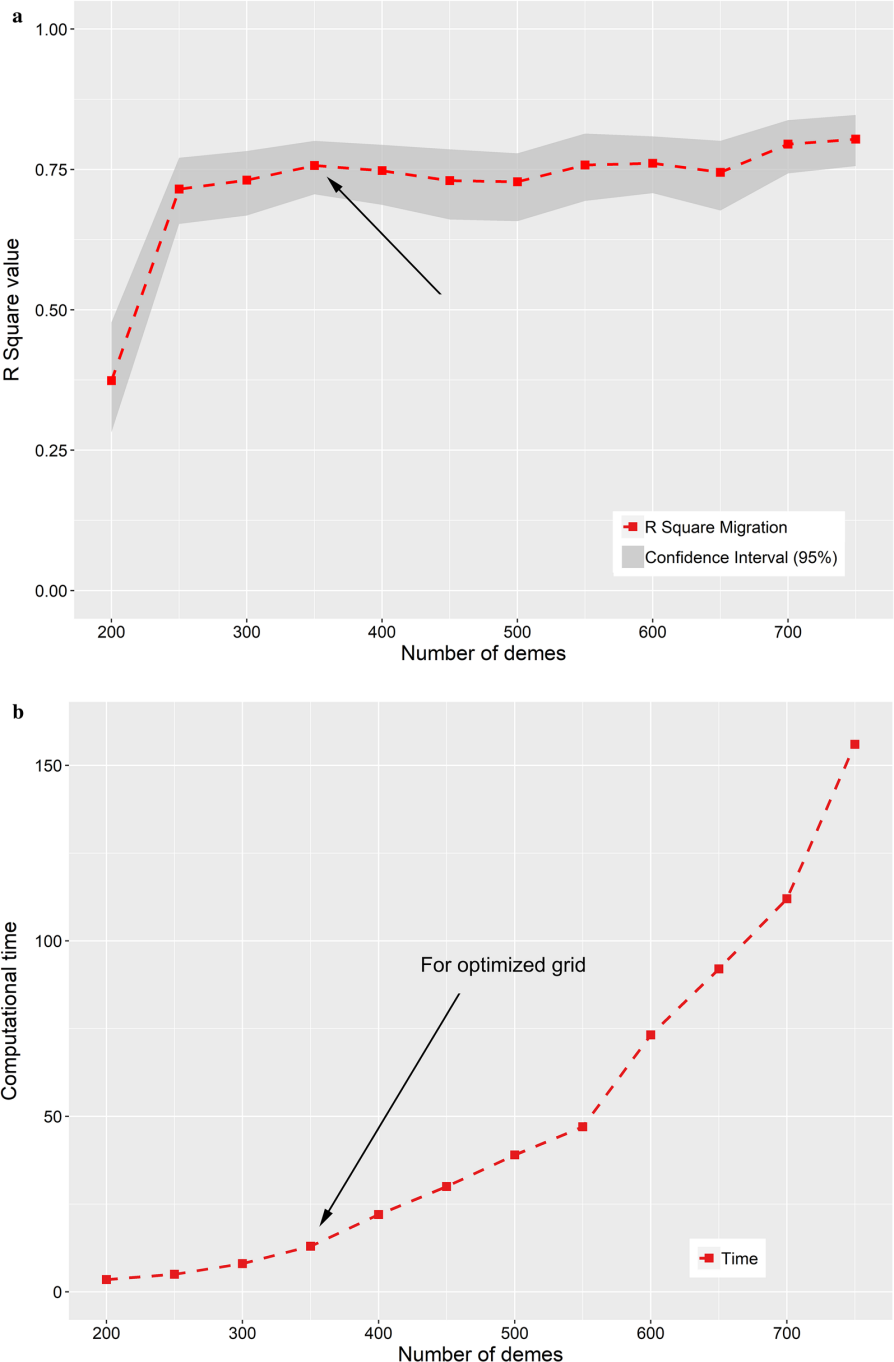
Model performance for 200 to 700 demes for **a** model accuracy (R^2^ value for migration model accuracy) and **b** computation time

To evaluate our optimization strategy, we used EEMS to generate migration surfaces for a range of grid resolutions from 200 to 750 demes, and compared the model accuracy and computing times for these grids with the optimized grid (Fig. 5). Using a grid of 200 demes had the poorest performance with R^2^ = 0.38 (Fig. 5a). A grid of 400 demes also had a slightly lower R^2^ value of 0.748 compared to the optimized grid (R2 = 0.757). And while R^2^ values appeared to increase for grids with more than 550 demes, these cases were associated with excessive computing times (Fig. 5b). The computing time for the 350-demes case (approximately 13 h) was much less than for 400 demes (28 h), 500 demes (39 h), 600 demes (73 h) and 700 demes (112 h). Running the analyses with different grid resolutions indicated that optimizing the number of demes offered the best performance for migration estimates with a significantly reduced computing time.

### Effective migration surfaces using the optimized grid

**Fig. 6:**
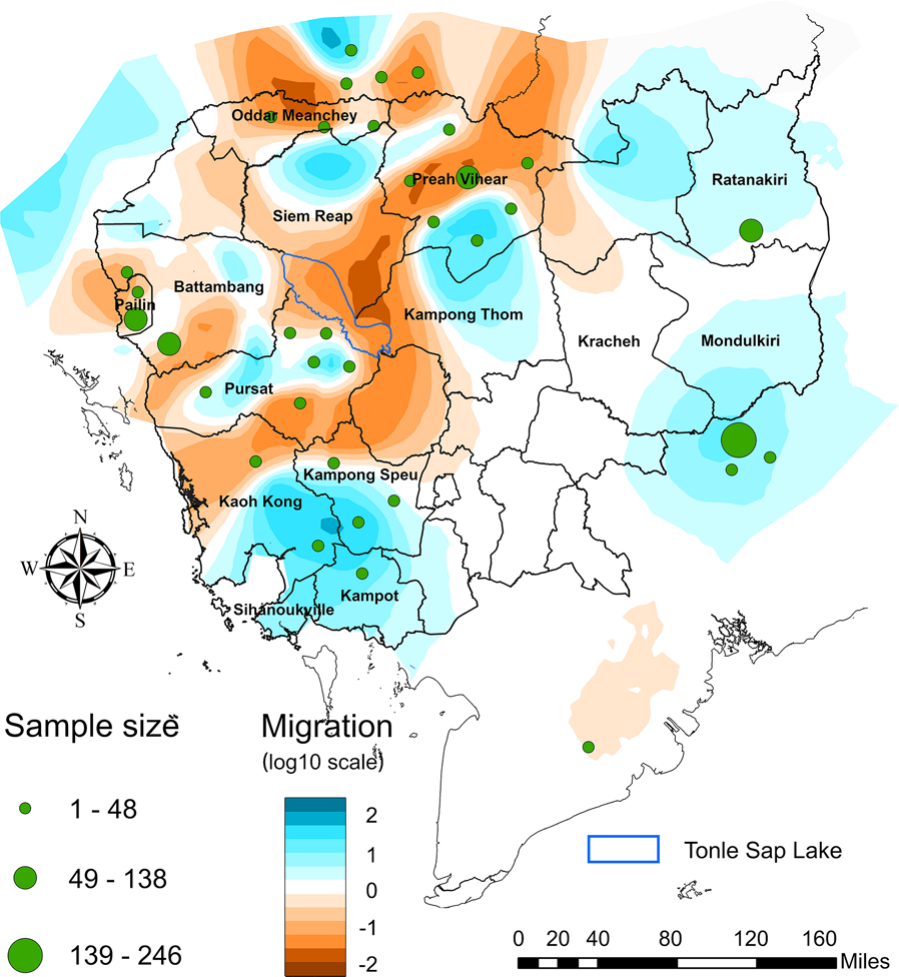
Estimated migration surface of *P. falciparum* parasites in Cambodia using the optimized grid

The optimized triangular grid was used to generate an estimated effective migration surface using parasite genomic data generated from isolates collected in Cambodia and surrounding locations. The migration contours in the resulting migration map (where blue indicates relative high migration and brown indicates lower migration) showed the lowest migration index value was near Tonle Sap Lake, which is the largest inland lake in Cambodia with an area of over 12,876 sq.km, while Koh Kong Province in the southwest showed the highest migration (Fig. 6). Southwest Cambodia (southern Koh Kong, southern Kampong Speu, Sihanoukville, Kampot, and Takeo Provinces) in general showed high migration relative to other locations, and the border area between eastern Cambodia and Vietnam was also associated with higher migration while locations in the border area of northwest Cambodia and Thailand showed lower parasite migration estimates.

### Comparison between estimated effective migration surfaces and *P. falciparum* endemicity and annual parasite incidence in Cambodia

**Fig. 7:**
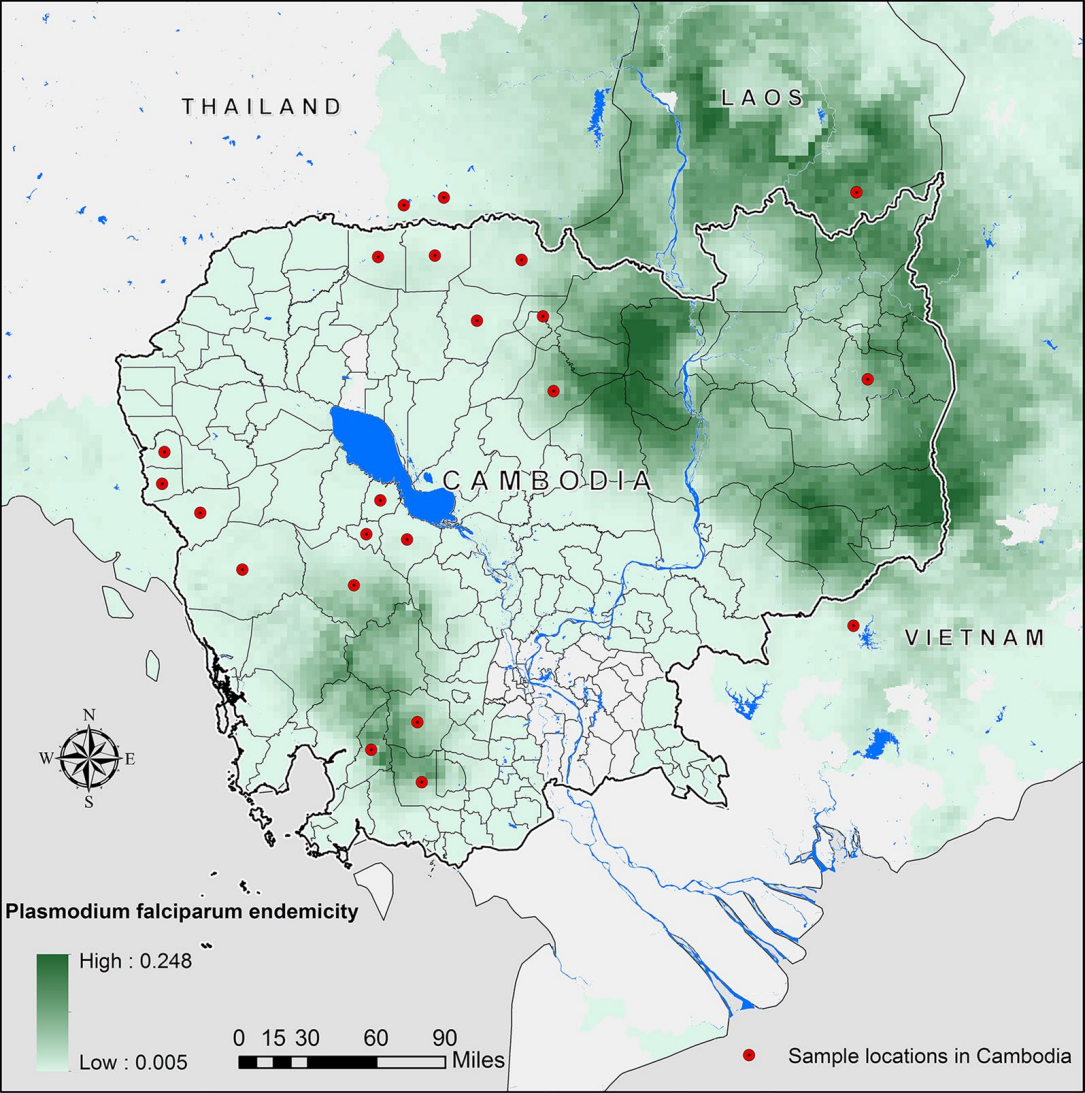
*P. falciparum* endemicity patterns within Cambodia from the Malaria Atlas Project (Data downloaded from https://map.ox.ac.uk/) (2010)

We compared the migration surface results generated using the spatially optimized grid (Fig. 6) with a *P. falciparum* endemicity map based on *P. falciparum* parasite rate (PfPR) data from 2010 made available through the Malaria Atlas Project [37] (Fig. 7), and also compared migration estimates with estimates of annual parasite incidence (API) per 1000 for 2013 [38]. The area of low migration near Tonle Sap Lake coincided with low P. *falciparum* endemicity. Areas with high *P. falciparum* migration in both southwestern and northeastern Cambodia were found to match regions with a relatively high prevalence of *P. falciparum*. This close relationship between *P. falciparum* migration and endemicity may imply the conditions in these locations are suitable for transmission of *P. falciparum* within these regions in Cambodia. High *P. falciparum* migration in northeastern Cambodia was consistent with high API values greater than 20% in the Steung Treng and Ratanakiri Provinces.

We also compared *P. falciparum* migration contours with data available from OpenDevelopment Cambodia on natural protected areas [39] and found that our results using EEMS corresponded to landcover features in a way we might expect, i.e., contours of high migration coincided with areas having mostly forest landcover (Additional file 1: Figure S1). For example, high *P. falciparum* migration in the northeastern and eastern regions of Cambodia (Fig. 7), corresponded to heavily forested areas, including a large National Park in Ratanakiri Province as well as four large wildlife sanctuaries in Mondulkiri and Ratanakiri provinces that could have served as habitats for *Anopheles* mosquitoes. Another area of high *P. falciparum* migration was located along the border between northwestern Cambodia and Thailand, and within northeastern Kampong Thom and south of Preah Vihear Province (Fig. 7) that were also forested with wildlife sanctuaries and protected natural habitats (Additional file 2: Figure S2). Another area of higher migration was observed in Battambang and Pursat provinces, just west of Tonle Sap Lake. Low migration patterns were more notable in western Cambodia, and a region of low *P. falciparum* migration extended from southern Laos, across the center of Cambodia, to southern Vietnam.

## Discussion

In this study, we developed a framework based on both genomic and spatial clustering to select the optimal number of demes to reduce spatial uncertainty in EEMS migration maps, in the presence of irregular sampling. EEMS migration contours can vary–sometimes substantially–based on the selected number of demes. Therefore, having a systematic, rational approach to determine grid density will likely be helpful to EEMS users. We were able to test and show that optimized grids displayed both high model accuracy and reduced processing time compared to grid densities selected in an unguided manner. In addition, when we utilized an optimized grid to generate EEMS migration maps for *P. falciparum,* we found that migration contours reflecting the parasite population structure corresponded to estimates of malaria endemicity and geographic properties of the study region (e.g., landcover and large waterbodies) that might be expected to impact malaria parasite migration.

The results of our genomic clustering approach indicated the presence of multiple genomic clusters based on malaria parasite genomic data generated from isolates collected in provinces in western, northwestern, and southwestern Cambodia. This finding is consistent with previous analyses of subsets of these data that found multiple sympatric genetic subpopulations of parasites that were hypothesized to have originated as founder populations resulting from the emergence of artemisinin resistance [40–42]. The congruence of these findings suggests that the density clustering approach applied in our optimization framework is accurately capturing known patterns of parasite genetic diversity in the study area.

Our results indicated overlap between areas of high *P. falciparum* migration and hotspots of malaria incidence in eastern Cambodia [43] [44–46], as well as other areas of high malaria endemicity [37, 47]. In Battambang and Pursat Provinces, high *P. falciparum* migration could result from flooding of the forests around Tonle Sap Lake during the wet season [48], providing habitats for malaria vectors [49]. In Pursat and Preah Vihear Provinces, high *P. falciparum* migration areas coincided with a high prevalence of multidrug resistance that is known to have emerged and spread in the area during this time frame [50].

Migration maps generated in this study corroborated major migration barriers for *P. falciparum* identified in our previous study [20]. However, use of the optimized grid allowed detection of a migration barrier in Pailin Province that was not identified in our previous analysis that is consistent with malaria elimination efforts in this area that have contributed to a dramatic decline in clinical malaria incidence [51–53]. *P. falciparum* migration barriers in northern Cambodia may have been due to higher urbanization (lower vegetation coverage) north of Tonle Sap Lake as well as Tonle Sap Lake itself, which is a large enough waterbody that it may have served as a barrier to *P. falciparum* migration. Deforestation of cardamom forests and large-scale land acquisitions in the area corresponded to the southern part of the ring-like contour of low migration and may also have been a contributor to this migration barrier [54, 55]. The Mekong River running through southeastern Cambodia as well as the urbanized area of Phnom Penh, may both have contributed to reduced parasite migration in this part of Cambodia. The fact that detected migration hotspots and barriers were geographically related with landcover and hydrologic features underscores the role that geography plays in shaping parasite population structure, which is consistent with findings from other studies, for example, a major migration barrier was detected around the Andes Mountains in Peru [56].

## Limitations and future work

Cambodia may be unique with respect to its patterns of parasite genetics due to multiple selection events of antimalarial drug resistance in the GMS region. This phenomenon is apparent through the overlapping genomic clusters, and ongoing work is addressing how selection of drug resistance may impact migration patterns observed in this area.

Future research will investigate how the spatial granularity of sampling may contribute to uncertainty in EEMS migration maps. For example, data from parasite isolates in this study were geolocated at the district level, which could lead to spatial uncertainty based on aggregation of multiple locations into a single location. Improving local geographical granularity may aid in detecting more detailed migration patterns. Further investigation is also required to improve our understanding of any boundary effects in EEMS analyses, as well as the impact of the assumption of geographic uniformity across a study area implied by use of a uniform grid, since geographic uniformity is not assumed for all studies [57], and is an assumption that is likely violated in many settings, including in studies of the malaria parasite as presented here.

## Conclusions

We have developed a semi-automatic workflow that used both genomic and spatial clustering to guide the optimization of triangle sizes and the density of demes, i.e., grid resolution, to generate effective migration surfaces for *P. falciparum* migration. Computing the analyses using different grid resolutions indicated that optimizing the number of demes offered the best performance for producing migration estimates with a significantly reduced computing time, an important consideration if maps are to be used to guide intervention strategies. We tested the optimized EEMS workflow on data generated from parasite isolates collected in Cambodia and bordering regions of Thailand and Vietnam, and found that migration contours corresponded to estimates of malaria endemicity and geographic features in the region that might be expected to impact malaria parasite migration (e.g., landcover, large waterbodies), supporting the validity of EEMS migration estimates. While in this study, our optimization framework was applied to malaria parasites, we believe this workflow is generalizable for other study areas and pathogens and can be used to guide the generation of migration maps based on available genomic sample distributions.

## Supplementary information

**Additional file****1****: Figure S1.** Optimized grid generated using the computed maximum triangle edge length.

**Additional file****2****: Figure S2.** Environmental features including protected areas, national parks, wildlife sanctuaries, and locations of waterbodies in Cambodia (2013).

## Data Availability

Genotyping data are publicly available through the MalariaGEN website (https://www.malariagen.net/data/p-falciparum-community-project-jan-2016-data-release) or through the NIH Gene Expression Omnibus (www.ncbi.nlm.gov/geo/) (Accession number: GSE100704) and European Variation Archive (Accession number: PRJEB28530) [20].

## Abbreviations

EEMS: Estimated effective migration surfaces
GMS: Greater Mekong Subregion
LISA: Local indicators of spatial autocorrelation
SNPs: Single nucleotide polymorphisms
MCMC: Markov chain Monte Carlo
P.f: *Plasmodium falciparum*
P.v: *Plasmodium vivax*
