# Supplementary figures and images for: Detecting geospatial patterns of Plasmodium falciparum parasite migration in Cambodia using optimized estimated effective migration surfaces
Source: Int J Health Geogr. 2020 Apr 10;19:13. doi: 10.1186/s12942-020-00207-3 (PMC7149848; doi:10.1186/s12942-020-00207-3)

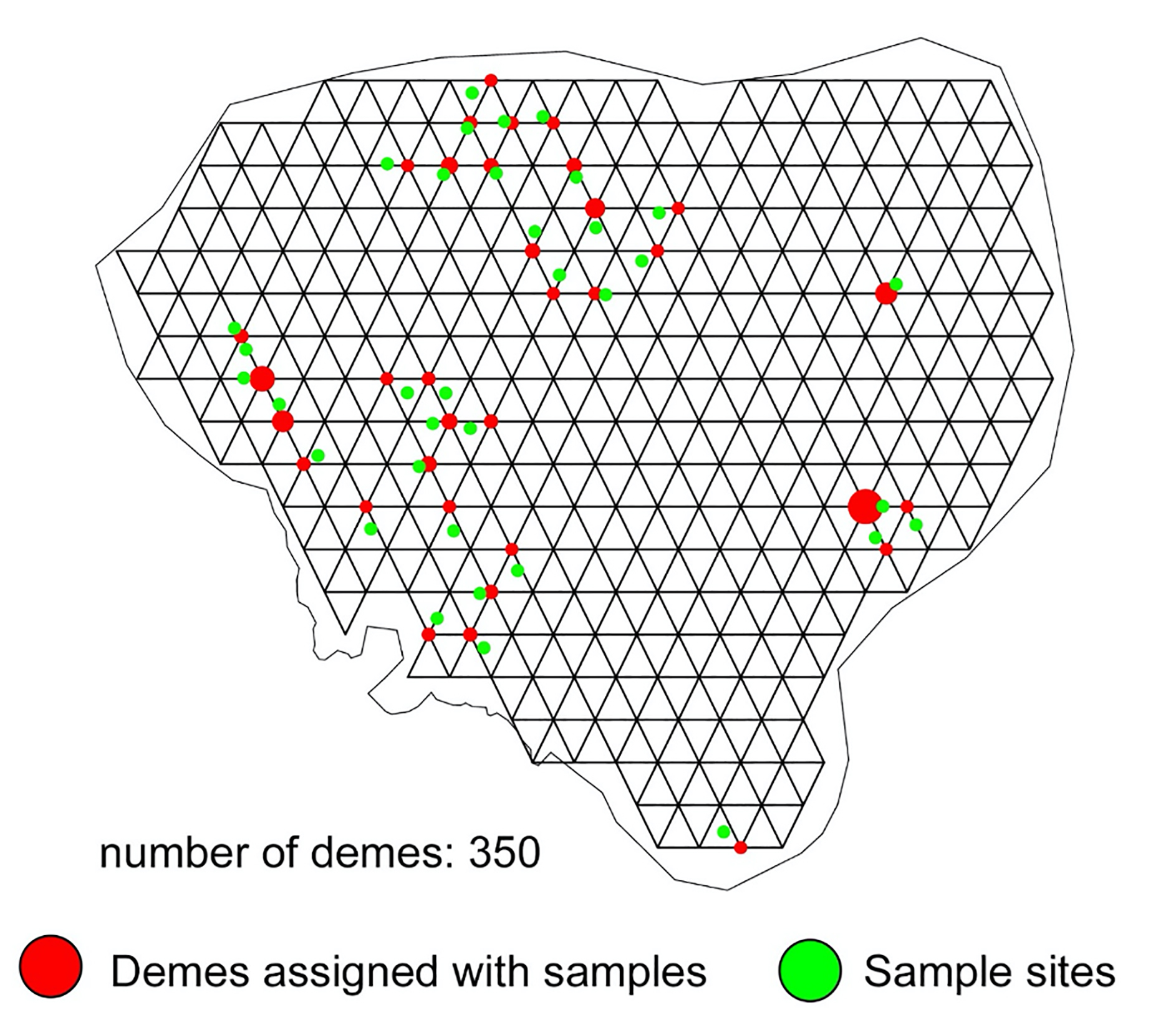

Supplement: Supplementary file 1 — Additional file1: Figure S1. Optimized grid generated using the computed maximum triangle edge length. [file 12942_2020_207_MOESM1_ESM.jpg]

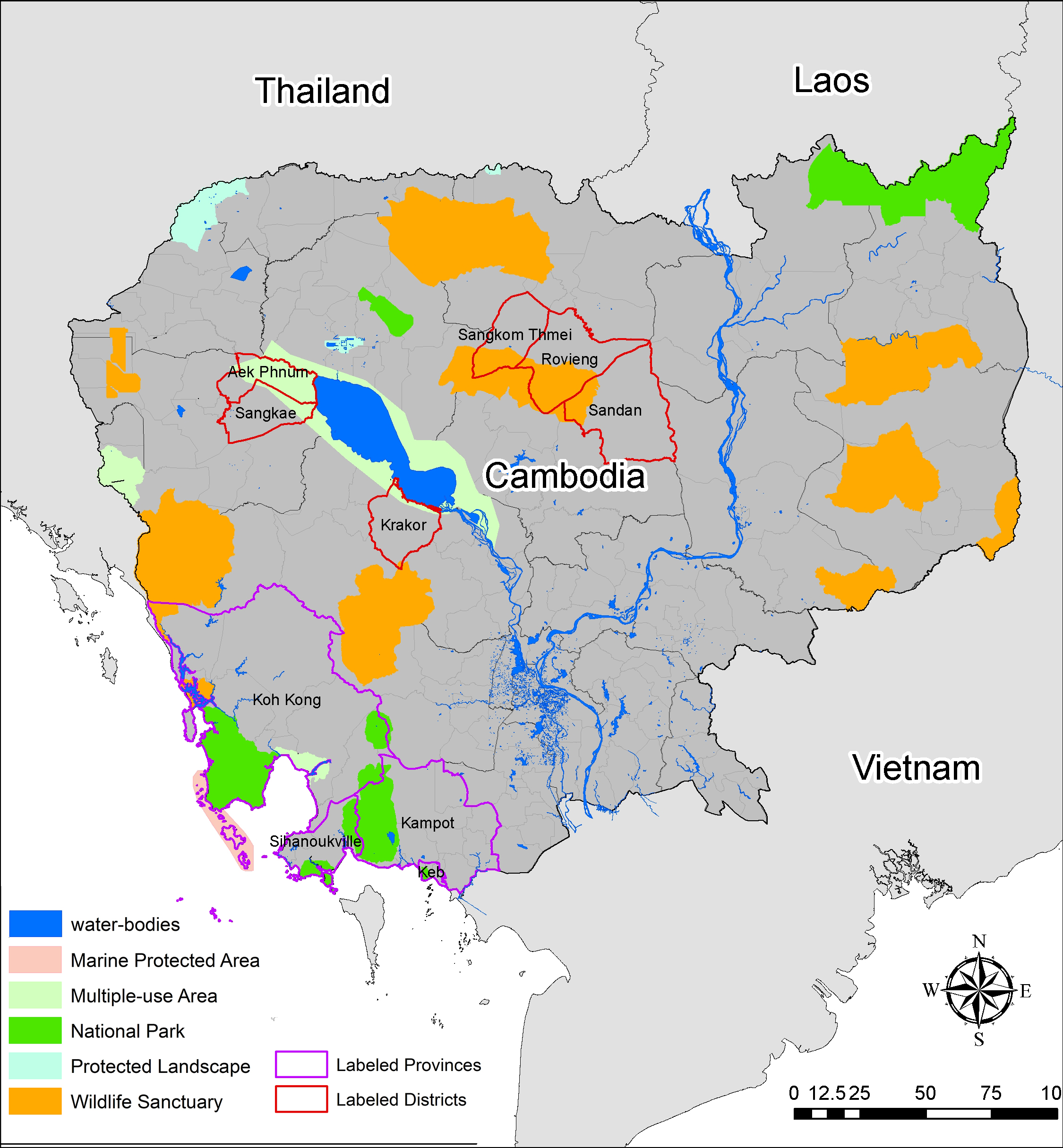

Supplement: Supplementary file 2 — Additional file2: Figure S2. Environmental features including protected areas, national parks, wildlife sanctuaries, and locations of waterbodies in Cambodia (2013). [file 12942_2020_207_MOESM2_ESM.jpg]
